# Cyclic Colour Change in the Bearded Dragon *Pogona vitticeps* under Different Photoperiods

**DOI:** 10.1371/journal.pone.0111504

**Published:** 2014-10-29

**Authors:** Marie Fan, Devi Stuart-Fox, Viviana Cadena

**Affiliations:** 1 Centre for Ecology and Conservation, College of Life and Environmental Sciences, University of Exeter, Penryn, United Kingdom; 2 Zoology Department, University of Melbourne, Parkville, Victoria, Australia; University of Texas Southwestern Medical Center, United States of America

## Abstract

The ability to change colour rapidly is widespread among ectotherms and has various functions including camouflage, communication and thermoregulation. The process of colour change can occur as an aperiodic event or be rhythmic, induced by cyclic environmental factors or regulated by internal oscillators. Despite the importance of colour change in reptile ecology, few studies have investigated the occurrence of a circadian rhythm in lizard pigmentation. Additionally, although colour change also entails changes in near-infrared reflectance, which may affect thermoregulation, little research has examined this part of the spectrum. We tested whether the bearded dragon lizard, *Pogona vitticeps*, displays an endogenous circadian rhythm in pigmentation changes that could be entrained by light/dark (LD) cycles and how light affected the relative change in reflectance in both ultraviolet-visible and near-infrared spectra. We subjected 11 lizards to four photoperiodic regimens: LD 12∶12; LD 6∶18; LD 18∶6 and DD; and measured their dorsal skin reflectance at 3-hour intervals for 72 hours after a habituation period. A proportion of lizards displayed a significant rhythm under constant darkness, with maximum reflectance occurring in the subjective night. This endogenous rhythm synchronised to the different artificial LD cycles, with maximum reflectance occurring during dark phases, but did not vary in amplitude. In addition, the total ultraviolet-visible reflectance in relation to the total near-infrared reflectance was significantly higher during dark phases than during light phases. We conclude that *P. vitticeps* exhibits a circadian pigmentation rhythm of constant amplitude, regulated by internal oscillators and that can be entrained by light/dark cycles.

## Introduction

Colour change is widespread in the animal kingdom, especially in ectothermic animals [1], including crustaceans [2], cephalopods [3], insects [4], amphibians [5], reptiles [6] and fishes [7]. The ability to modify skin coloration allows animals to accommodate the different demands of inter- or intra-specific communication [8], camouflage [9], thermoregulation [10], [11] and protection from the damaging effects of ultraviolet (UV) radiation [12]. At the proximate level, colour change is mediated by chromatophores (pigment cells that originate from the neural crest and migrate to the integument) and can be classified as two types [1]. Morphological colour change results from changes in the amount of pigment and/or the number of chromatophores and takes place over a timescale of days or months [1]. Physiological colour change is induced by the migration of pigment-containing organelles within chromatophores and is much more rapid, occurring in a matter of seconds to hours. Physiological colour change can occur either as a singular event or a cyclic event (i.e. display rhythmicity) which may be elicited by environmental stimuli or mediated by neural and/or endocrine systems [1]. Although adaptive colour change that occurs in response to specific stimuli (e.g. predators, conspecifics) has been widely studied, the occurrence and regulation of rhythmic colour change has received much less attention.

Rhythmic physiological colour change has been reported in both invertebrates [2], [13], [14] and vertebrates [15]–[17] and may be of either exogenous or endogenous nature. Exogenous rhythms are induced by rhythmic environmental stimuli whereas endogenous rhythms rely on biological oscillators and allow the animals to anticipate and adapt to cyclic environmental events and thus reduce costs induced by potential delays when relying on external cues. The most conspicuous biological rhythms are circadian rhythms that continue to oscillate even under constant conditions with periods close to 24 hours and are caused by multiple internal oscillators [18], [19]. However, these rhythms are also sensitive to external entrainment cues or “zeitgebers” that trigger and synchronise them with the 24-hour rhythm of the Earth’s rotation [18]. The relative dominance of endogenous and exogenous stimuli in synchronising each independent oscillator is unclear; yet social and environmental factors, such as light, temperature or humidity, are undoubtedly important [2].

Most reported cases of circadian colour change concern crustaceans, especially crabs [2], [14], [20], [21]. Light/dark (LD) cycles have also been reported to play a crucial role in circadian pigmentation rhythms [2], particularly in the toad *Bufo ictericus* [17] and the fiddler crab *Uca panacea* [14]. However, despite the important role of colour change in the behavioural ecology of reptiles, few studies have investigated the existence of a circadian rhythm of colour change in this taxonomic group. Caswell (1950) detected the occurrence of a daily colour change in the lizard *Xantusia vigilis* [15] and Underwood (1985) and Binkley *et al.* (1987) examined the role of pineal melatonin in rhythmic colour change in the lizard *Anolis carolinensis* [22], [23]. Nevertheless, there is very little information regarding the mechanisms underlying such a rhythm, including potential entrainment cues, and its functional significance.

Most studies of colour change have focused on the visible spectrum of solar radiation (400–700 nm) and, more recently, on the UV spectrum (especially the near-UV spectrum; 300–400 nm) to which the vision of many species is sensitive. However, surfaces reflect a much broader spectrum of solar radiation, which ranges from 290 to 2500 nm. Although no animal can visually detect reflected radiation in the near-infrared range (NIR; 700–2150 nm), reflectance in this part of the spectrum is critical for thermoregulation because more than half of the sun's energy-rich radiation falls within this range [24]. Colour change in the UV-visible (UV-Vis) spectrum necessarily entails a change in NIR reflectance because they are not independent (i.e. reflectance is continuous across the full spectrum of solar radiation); however relative changes in UV-Vis and NIR reflectance have rarely been characterised. Therefore, to fully characterise and understand circadian changes in reflectance, it is important to measure spectral change both within the UV-Vis and NIR ranges.

The central bearded dragon lizard *Pogona vitticeps* (Ahl 1926) is a good biological model for the examination of a circadian pigmentation rhythm because of (1) its ability to exhibit marked colour change; (2) high inter-individual variation in colouration; and (3) its use of colour change for visual signalling and thermoregulation [25]. In the wild, the species inhabits semiarid to arid woodlands and rocky desert regions and is active during the day (except when hibernating) spending the morning and early evening basking on branches or on the ground and retreating to shady areas when temperatures are the highest. As far as we know, however, no study has established the existence of a circadian colour change in this species. We examined this process in a captive population of *P. vitticeps* originating from the vicinity of Alice Springs, Australia, where day length ranges from 10.5 to 13.5 hours. More specifically, this study (1) determined whether *P. vitticeps* displays an endogenous pigmentation rhythm with a circadian period; (2) tested whether this rhythm can be entrained by light/dark cycles; (3) characterised the extent of colour change within such a rhythm; and (4) examined how light affects the relative change in UV-visible (300–700 nm) and NIR (700–2150 nm) reflectance.

## Materials and Methods

### Animals

Adult males of *P. vitticeps* (n = 11) were collected near Alice Springs (23°42′S, 133°52′E) between late September and early October 2012 and brought to an indoor animal facility at the Zoology Department, The University of Melbourne. Lizards were housed individually in terraria fitted with a UV lamp and a heat lamp set to a light/dark (LD) 12∶12 photoperiod (lights on at 07∶00 and off at 19∶00). Each terrarium contained a hiding place, a perch and sand substrate. The temperature inside the terraria ranged from ∼25°C under the hiding place to ∼50°C directly under the basking light, allowing for behavioural thermoregulation. Animals were provided water *ad libitum* and fed a diet of chopped green leafy vegetables, carrots and pumpkins, bearded dragon pellets (Ultimate Reptile Supplies, Australia) and crickets three times a week.

### Ethics Statement

The study did not involve endangered species. It was carried out on captive lizards at the University of Melbourne. Animals were collected from the wild (GPS coordinates: 23°42′S, 133°52′E) under relevant permits listed below. The permits for collecting and importing the lizards were provided by (1) the Victorian Department of Environment and Primary Industries (Scientific Permit Number: 10006453; Import Permit Number: 14237999); (2) the Northern Territory Parks and Wildlife Commission (Export Permit Number: 45795; Collection Permit Number: 44582); (3) the Northern Territory Central Land Council for entering and remaining on Aboriginal land (Permit Number: 15396) and (4) the Northern Territory Department of Lands and Planning for collecting on roadsides (Permit Number: DDPI 2009/7281∼0013). All experimental procedures were approved by the Animal Ethics Committee of The University of Melbourne (Permit Number: 1212547). They were not invasive, involved only minimal handling and therefore involved no pain and minimal stress.

### Experiments

Experiments were conducted in the Austral autumn from April 3 to June 3, 2014. To examine circadian colour change, the 11 individuals were subjected to four photoperiodic regimens: (1) LD 12∶12 (lights on at 07∶00 and off at 19∶00); (2) LD 6∶18 (lights on at 10∶00 and off at 16∶00); (3) LD 18∶6 (lights on at 04∶00 and off at 22∶00) and (4) DD (lights remained off). Therefore, the light portion of LD 6∶18 and LD 18∶6 was shortened and extended compared to LD 12∶12, but the midpoint of the light phase was always at the same hour (13∶00) whereas the onset of light shifted in time. Experiments were conducted in a constant temperature room set to 30°C and lit during the light phase with three pairs of 36 W fluorescent lights (L 36W/830, ‘warm daylight’, Osram, Germany) combined with 26 W UVB lights (Exo Terra Reptile UVB150, Exo Terra, USA) to provide full-spectrum lighting approximating the spectral range of daylight. The lights were suspended 2 m above plastic tubs (65 cm length×42 cm width×39 cm height). The total light intensity in the room, measured with an Extech environmental meter 4517 at the height of the lizards, was approximately 400 lux. The 11 lizards were divided into two groups (n = 5 and n = 6) and each group was subjected to each of the four different photoperiodic regimens in random order with at least six days of rest in their home terraria (under a LD 12∶12 regimen) between two successive experiments.

**Figure 1:**
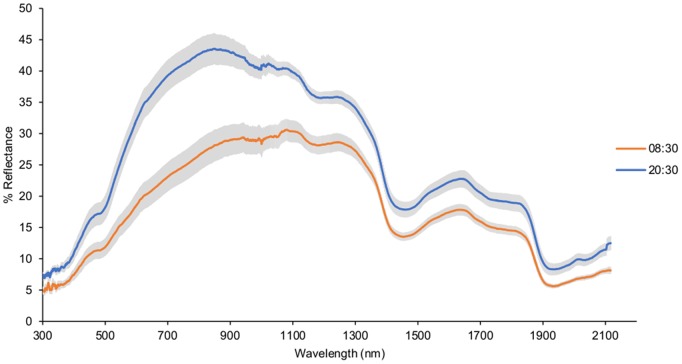
Dorsal skin reflectance of rhythmic lizards under LD 12∶12, measured during the light and the dark phases. Reflectance (expressed in %) was measured from 300 to 2150 nm at 08∶30 during the light phase (orange curve) and at 20∶30 during the dark phase (blue curve) on the same day. Reflectance is expressed as mean ± s.e.m (grey area) for all the rhythmic lizards under LD 12∶12 (n = 7).

For each experiment, the lizards were randomly and individually placed in the tubs and were acclimated in the experimental room for a day when exposed to the LD 12∶12 regimen (as they were already experiencing this regimen) and for 3 days when exposed to the three other regimens. Lizards were misted and fed with 5–6 crickets on the last day of acclimation. After the phase of acclimation, the reflectance from 300 to 2150 nm of each lizard’s dorsal skin was measured using two spectrometers (USB2000+ and NIRQuest, Ocean Optics, USA) at 3-hour intervals starting from 08∶30 for 72 hours (Figure 1; also see Figure S1). The spectrometers were calibrated against a 99% white diffuse reflectance standard (WS-1-SL, Ocean Optics, USA). The measurements were done with a flat probe (RPH-1, Ocean Optics, USA) placed against each lizard’s skin, in the middle of the back. A 15 W incandescent red lamp (Cat. No.: 15W red globe, GMT/Clipsal, Australia) was turned on for the measurements done during the dark phase. The light intensity of the red lamp at the height of the lizards was 0 lux. Reflectance values were recorded using the software OceanView (Ocean Optics, USA).

### Data analysis

**Figure 2:**
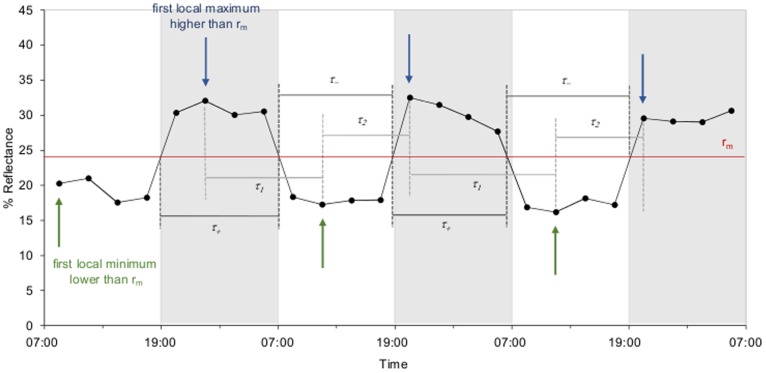
Average dorsal skin reflectance of a rhythmic lizard exemplifying the parameters characterising the rhythms. The lizard was exposed to LD 12∶12. Reflectance (expressed in %) was averaged for 300–2150 nm. The red line corresponds to the average reflectance (r_m_). Blue arrows indicate first local maxima higher than r_m_; green arrows indicate first local minima lower than r_m_. The graph shows how *τ_1_*, *τ_2_*, *τ_+_* and *τ_-_* were calculated for two complete reflectance cycles (here starting from 23∶30 for *τ_1_* and *τ_2_* and from 19∶00 for *τ_+_* and *τ_-_*) before being averaged. *τ_1_* represents the time interval between a first local maximum reflectance higher than the average reflectance and the following first local minimum reflectance lower than the average reflectance, *τ_2_* the time interval between a first local minimum reflectance lower than the average reflectance and the following first local maximum reflectance higher than the average reflectance, *τ_+_* the time interval wherein the reflectance is higher than the average reflectance and *τ_-_* the time interval wherein the reflectance is lower than the average reflectance. White and grey bars indicate respectively the light and dark phases experienced by the lizard.

To test the existence of a circadian pigmentation rhythm, the average reflectance from 300 to 2150 nm was calculated for each reflectance measurement (Figure 2). Time series of average dorsal skin reflectance were then analysed for periodicity using autocorrelation and maximum entropy spectral analysis (MESA) for each individual. MESA fits an autoregressive model to the data and uses Fourier analysis to construct a power spectrum from which period estimates can be obtained [26]. Rhythmicity (i.e. the presence of a rhythm) was assessed using autocorrelation analysis which provided correlograms where autocorrelation coefficients were plotted as a function of lag at 3-hour intervals [27]. Several lizards displayed obvious rhythmicity at a *p* value between 0.1 and 0.15, as validated by the correlograms, but did not reach the significance threshold of *p*<0.05 due to the moderate sampling rate. Therefore, we considered that time series which showed significant rhythmicity at *p*<0.15 and for which MESA provided a period length estimate close to 24 hours, validated by comparison with correlograms, could be reasonably considered as displaying a rhythm. By increasing the significance threshold to *p*<0.15, we also wished to avoid the risk of rejecting individuals displaying a rhythmicity only because of the moderate sampling rate. Peaks in the correlogram with a coefficient exceeding ±1.44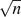, where *n* is the number of 3-hour intervals in the time series (*n* = 24), indicated statistically significant rhythmicity at *p*<0.15. Period length estimates (*τ*) were obtained using MESA and validated by comparison with peaks in the correlograms. In the absence of such peaks in agreement with period length estimates, the time series was considered as arrhythmic and removed from the rest of the analysis. Lizards displaying significant rhythmicity in dorsal skin reflectance will be referred as “rhythmic lizards”. For all the rhythmic lizards, the period length estimates obtained using MESA were close to 24 hours, with mean values +/− one hour of 24 hours (Table 1). We tested whether the estimated period length differed significantly from the expected value of 24 hours using a one sample *t*-test for each photoperiodic regimen. The estimated period lengths were not significantly different from 24 hours for any light regimen (DD: *t*_4_ = 0.03, *p* = 0.98; LD 12∶12: *t*_6_ = 0.96, *p* = 0.38; LD 6∶18: *t*_4_ = 0.81, *p* = 0.46; LD 18∶6: *t*_6_ = −1.62, *p* = 0.16).

**Table 1:** Summary of results for the four photoperiodic regimens for lizards displaying significant rhythmicity.

Photoperiodic regimen	Percent rhythmic (%)	*τ* (h)	*φ* (h)	r_m_ (% reflectance)	A(r) (% reflectance)	*δ*_m_ (%)	A*(δ)* (%)
DD	45.5	24.0±0.9	21∶06±0.5	23.7±0.8	9.9±1.4	40.4±0.6	10.5±1.4
LD 12∶12	63.6	24.5±0.5	00∶21±0.5	24.5±1.2	12.3±1.0	39.4±1.8	9.8±1.5
LD 6∶18	45.5	25.0±1.1	21∶06±1.0	22.9±1.1	12.1±1.1	40.6±1.7	9.3±1.8
LD 18∶6	63.6	23.1±0.5	02∶04±0.7	24.5±0.7	12.0±0.4	39.8±1.6	10.0±1.1
**Photoperiodic regimen**	***τ*** **(** ***φ*** **) (h)**	**Δ(** ***φ*** **) (h)**	***τ_1_*** ** (h)**	***τ_2_*** ** (h)**	***τ_+_*** ** (h)**	***τ_−_*** ** (h)**	
DD	14.1±0.5	NA	10.5±1.5	13.2±1.0	11.0±0.2	13.4±0.4	
LD 12∶12	17.4±0.5	5.4±0.5	11.4±0.7	12.2±0.6	11.3±0.3	12.4±0.4	
LD 6∶18	14.1±1.0	5.1±1.0	15.6±0.9	6.3±1.0	13.9±1.0	10.1±1.1	
LD 18∶6	19.1±0.7	4.1±0.7	7.1±1.1	16.3±1.3	8.8±0.7	14.9±0.9	

Percent rhythmic indicates the percentage of rhythmic lizards. *τ* represents the period, *φ* the acrophase, r_m_ the average reflectance, A(r) the reflectance amplitude, *δ*_m_ the average standardised difference between the total reflectance in near-infrared (700–2150 nm) and the total reflectance in ultraviolet-visible (300–700 nm), A(*δ*) the amplitude of *δ*, *τ*(*φ*) the time interval from 07∶00 to the acrophase, Δ(*φ*) the time interval from the beginning of the dark phase to the acrophase, *τ_1_* the average time interval between the first local maximum reflectance higher than the average reflectance and the following first local minimum reflectance lower than the average reflectance, *τ_2_* the average time interval between the first local minimum reflectance lower than the average reflectance and the following first local maximum reflectance higher than the average reflectance, *τ_+_* the average time interval wherein the reflectance is higher than the average reflectance and *τ_-_* the average time interval wherein the reflectance is lower than the average reflectance. All the parameters are expressed as mean ± s.e.m. NA: not applicable.

To test whether the circadian pigmentation rhythm of rhythmic lizards was entrained by light, several parameters characterising the reflectance variations over a cycle were calculated. Cross-correlation analysis [27] was used to estimate the acrophase (i.e. the time of the day where the reflectance was maximum; *φ*). For all the experiments using a photoperiodic regimen other than DD, the 24-hour diel cycle was represented by an arbitrary sine curve with a maximum value of 1 at 07∶00 and a minimum value of −1 at 19∶00. For the experiment using DD, we used an arbitrary sine curve with a period length equal to the one provided by MESA for each rhythmic lizard and with a maximum value of 1 at 07∶00. Cross-correlation coefficients were plotted as a function of lag at 3-hour intervals and peaks between 0 and 24 h indicated the time of maximum reflectance relative to the arbitrary sine curve. To be able to perform a quantitative analysis on *φ*, two additional parameters were calculated: (1) the time interval from 07∶00 to the acrophase (*τ*(*φ*)) and (2) the time interval from the beginning of the dark phase to the acrophase (Δ(*φ*)).

To examine whether the dorsal skin reflectance was responsive to the length of both light and dark phases, four parameters were calculated (Figure 2): (1) the time interval between a first local maximum higher than the average reflectance r_m_ and the following first local minimum lower than r_m_ (*τ_1_*); (2) the time interval between a first local minimum lower than r_m_ and the following first local maximum higher than r_m_ (*τ_2_*); (3) the time interval wherein reflectance values were higher than r_m_ (*τ_+_*); and (4) the time interval wherein reflectance values were lower than r_m_ (*τ_-_*). Using the first local extremes enabled us to follow the reflectance variations when switching from one phase to another and referring to the average reflectance reduced the risk of using local extremes which were most likely artefacts (i.e. random peaks occurring within a given phase). In addition, using the time intervals wherein reflectance was higher or lower than the average value provided supplementary information regarding the shape of the curve (e.g. the time interval *τ_+_* differs when the reflectance is relatively constant during the dark phase compared to when it is decreasing during the dark phase). Each of these parameters was extracted from two complete reflectance cycles and averaged over these two cycles (Figure 2). We only used two cycles instead of three because the calculation was based on the intersections of the reflectance curve with the line of average reflectance r_m_. The first couple of intervals wherein reflectance was first higher than r_m_ then lower than r_m_ constituted the first complete cycle; the second couple of intervals wherein reflectance was first higher than r_m_ then lower than r_m_ constituted the second complete cycle (Figure 2).

To assess the extent of colour change in the circadian pigmentation rhythm, reflectance amplitudes (i.e. the difference between the reflectance extreme values; A(r)) were extracted from each 24-hour cycle of the time series and averaged over the three cycles.

Finally, to assess the relationship of reflectance in the ultraviolet-visible (UV-Vis; 300–700 nm) region in relation to the near-infrared (NIR; 700–2150 nm) region, the standardised difference between the total reflectance in NIR and the total reflectance in UV-Vis (*δ*) was calculated using the following formula:
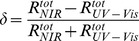
(1)where 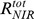 is the total reflectance in the NIR region; and


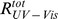
 is the total reflectance in the UV-Vis region.

Average values and amplitudes of *δ* (respectively *δ*_m_ and A(*δ*)) were calculated for each 24-hour cycle of the time series and averaged over the three cycles. In addition, to determine whether *δ* differed between light and dark phases, *δ* values respectively at maximum reflectance and minimum reflectance were extracted from each 24-hour cycle and averaged over the three cycles.

One-way analyses of variance (ANOVAs) were used to test for significant variation due to photoperiod for parameters characterising the displayed rhythm. Tukey’s Honestly Significant Difference (HSD) test was used for post hoc pairwise comparisons in order to test for differences between the four photoperiodic regimens. A paired *t*-test was used to test for significant differences in *δ* values between light and dark phases.

All analyses were done using Microsoft Excel 2013, R version 3.0.2 (R Core Team 2013) and executable files provided by Harold B. Dowse for the autocorrelation analysis, MESA and cross-correlation analysis [28].

## Results

### Circadian rhythm in dorsal skin reflectance under constant darkness

**Figure 3:**
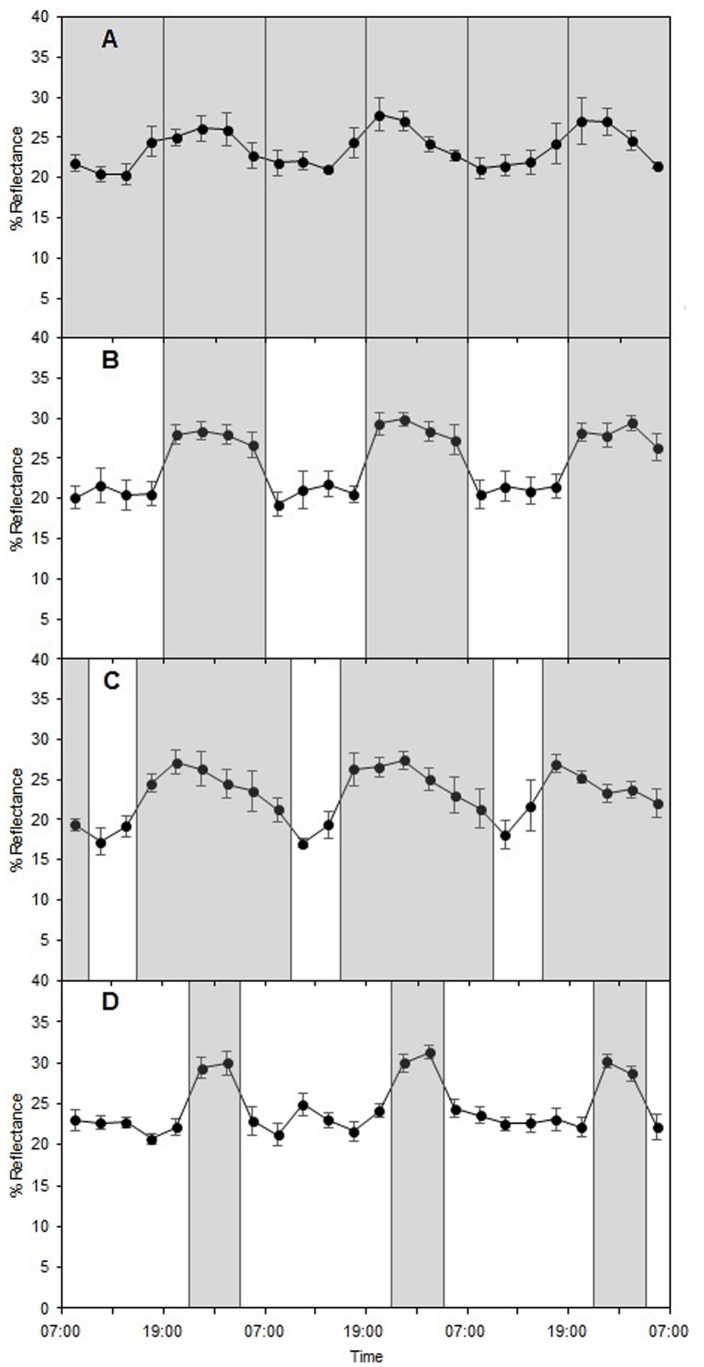
Average dorsal skin reflectance of lizards displaying significant rhythmicity under the four photoperiodic regimens. Reflectance (expressed in %) was measured under (A) DD (n = 5 lizards), (B) LD 12∶12 (n = 7), (C) LD 6∶18 (n = 5) and (D) LD 18∶6 (n = 7) and averaged for 300–2150 nm. Reflectance measurements started after 1 day of exposure for LD 12∶12 and after 3 days of exposure for the other photoperiodic regimens**.** White and grey bars indicate respectively the light and dark phases experienced by the lizards. Reflectance is expressed as mean ± s.e.m.

Five of the 11 lizards (45.5%) displayed clear rhythmicity (*p*<0.15) under constant darkness (DD), with a period length of 24.0±0.9 h (mean ± s.e.m; Table 1), suggesting the existence of an endogenous circadian rhythm. The reflectance variations – reflected by the parameters *τ_1_*, *τ_2_*, *τ_+_* and *τ_-_* (Figure 2) – were approximately symmetrical around the mean (*τ_1_* = 10.5±1.5 h and *τ_2_* = 13.2±1.0 h; *τ_+_* = 11.0±0.2 h and *τ_-_* = 13.4±0.4 h; Table 1), corresponding to a sine-type curve (Figure 3A). In addition, the acrophase (i.e. the time of the day where the reflectance was maximum) occurred at 21∶06±0.5 h (Table 1), indicating that under constant darkness, the lizards’ dorsal skin became the lightest in the subjective night.

### Daily rhythm in dorsal skin reflectance under artificial light/dark cycles

Lizards were exposed to three artificial LD cycles: (1) LD 12∶12; (2) LD 6∶18 and (3) LD 18∶6. A proportion of lizards showed clear rhythmicity (*p*<0.15) under all three artificial LD cycles (LD 12∶12: 63.6%; LD 6∶18: 45.5% and LD 18∶6: 63.6%; Table 1). Under all three regimens dorsal skin was lighter during the dark phases and darker during the light phases (Figure 3). Unlike the period length, which did not differ between photoperiodic regimens (ANOVA, F_3,20_ = 1.04, *p* = 0.40), the acrophase (more precisely the time interval from 07∶00 to the acrophase; *τ*(*φ*)) differed significantly (ANOVA, F_3,20_ = 10.24, *p*<0.001). Post hoc pairwise comparisons showed that the acrophase differed between all regimens except between LD 6∶18 and DD and between LD 18∶6 and LD 12∶12 (Table S1). The shift in rhythm in response to photoperiod was confirmed by significant differences between regimens in 5 parameters (*τ*(*φ*), *τ_1_*, *τ_2_*, *τ_+_* and *τ_-_*) characterising the displayed rhythms (Table 2). Specifically, the acrophase was significantly delayed in the 18∶6 compared to the 6∶18 photoperiod (Tukey’s HSD test, *p*<0.01), suggesting an entrainment by light because the acrophase always occurred within the same time range after the lights went out (Tables 1, 2). Furthermore, the significantly lower *τ_1_* and *τ_+_* values (Tukey’s HSD test, *p*<0.001 for both parameters) and significantly higher *τ_2_* and *τ_-_* values (Tukey’s HSD test, *τ_2_*: *p*<0.001; *τ_-_*: *p*<0.01) in the 18∶6 compared to the 6∶18 photoperiod show that the pigmentation rhythm synchronised to the length of the light and dark phases (Figure 3C,3D).

**Table 2:** Summary of results provided by a one-way ANOVA used to assess the effect of the photoperiodic regimen on dorsal skin reflectance of rhythmic lizards.

One-way ANOVA						
Parameter	*τ*	r_m_	A(r)	*δ*_m_	A(*δ*)	
F value	F_3,20_ = 1.04	F_3,20_ = 0.46	F_3,20_ = 0.96	F_3,20_ = 0.10	F_3,20_ = 0.09	
*p* value	0.40	0.71	0.43	0.96	0.97	
Parameter	*τ*(*φ*)	Δ(*φ*)	*τ_1_*	*τ_2_*	*τ_+_*	*τ_−_*
F value	F_3,20_ = 10.24	F_2,16_ = 0.79	F_3,20_ = 9.06	F_3,20_ = 13.81	F_3,20_ = 9.68	F_3,20_ = 5.78
p value	***	0.49	***	***	***	**

F values and *p* values are provided for parameters characterising the reflectance rhythm (*τ*, r_m_, A(r), *δ*_m_, A(*δ*), Δ(*φ*), *τ*(*φ*), *τ_1_*, *τ_2_*, *τ_+_* and *τ_-_* which are defined in the materials and methods section and in Table 1). *p<0.05; **p<0.01 and ***p<0.001.

### Extent of circadian colour change

Average reflectance did not significantly differ under the four different photoperiodic regimens (ANOVA, F_3,20_ = 0.46, *p* = 0.71), varying from 22.9±1.1% to 24.5±1.2% (Tables 1, 2; also see Figure S2). Similarly, the amplitude of the reflectance rhythm was not significantly affected by the different photoperiodic regimens (ANOVA, F_3,20_ = 0.96, *p* = 0.43), varying from 9.9±1.4% to 12.3±1.0% (Tables 1, 2; also see Figure S2). In addition, although the average reflectance did not vary much among individuals, the amplitude of the rhythm was quite variable, ranging from 6.3% for the smallest recorded amplitude to 15.8% for the highest, with some lizards displaying clearly noticeable skin darkening during the light phase and skin lightening during the dark phase.

### Relationship between reflectance in UV-Vis and NIR

The average standardised difference between the total reflectance in NIR and the total reflectance in UV-Vis (*δ*) did not significantly differ under the different photoperiodic regimens (ANOVA, F_3,20_ = 0.10, *p* = 0.96), varying from 39.4±1.8% to 40.6±1.7% (Tables 1, 2; also see Figure S3). Similarly, the amplitude of *δ* did not significantly differ under the different photoperiodic regimens (ANOVA, F_3,20_ = 0.09, *p* = 0.97), varying from 9.3±1.8% to 10.5±1.4% (Tables 1, 2; also see Figure S3). Again, the average *δ* did not vary much among individuals, but the amplitude of *δ* was quite variable – ranging from 3.7% for the smallest recorded amplitude to 16.1% for the highest. Interestingly, these extreme values of A(*δ*) do not correspond to those of A(r), indicating that lizards which show a similar extent of reflectance change may differ in the ratio of total reflectance in NIR and UV-Vis. Additionally, lizards showed relatively greater circadian change in the UV-Vis than NIR spectrum such that higher reflectance during the dark phase entailed a relatively higher proportion of UV-Vis than NIR reflectance (as indicated by significantly lower *δ* values) compared to the lower overall reflectance during light phases (paired *t*-test, *t* = −6.46, df = 18, *p*<0.001).

## Discussion

This study investigated the circadian pigmentation rhythm in the central bearded dragon lizard *P. vitticeps*. A proportion of lizards (45.5%) displayed an endogenous circadian rhythm in dorsal skin reflectance, oscillating between a maximum occurring in the subjective night and a minimum occurring in the subjective day. Such a rhythm was also exhibited in a proportion of lizards exposed to artificial light/dark cycles which entrained this rhythm – their skin being darker during light phases and lighter during dark phases – without affecting the extent of colour change. In addition, the total reflectance in UV-Vis in relation to the total reflectance in NIR was higher during dark phases than during light phases, but also varied in amplitude among the lizards. Therefore, *P. vitticeps* seems to have one or more internal oscillators inducing a circadian pigmentation rhythm of constant amplitude that can be entrained by light/dark cycles and responds to light by a decrease in skin reflectance, this decrease being proportionately larger in the UV-Vis range than in the NIR range.

Although rhythmic colour change in lizards has been reported in the lizards *X. vigilis* [15] and *A. carolinensis* [22], [23], [29], little research has been undertaken to characterise this process. To our knowledge, this is the first study that provides insight into circadian reflectance change in lizards across the great majority of the solar spectrum (300–2150 nm) and examines the role of light as an entrainment cue. Similar studies have been carried out for the visible spectrum on the toad *B. ictericus* [17] and on the crab *U. panacea* [14] and both reported the presence of a circadian colour change responsive to light cues.

Our results establish the existence of an endogenous circadian rhythm in the dorsal skin reflectance of the bearded dragon *P. vitticeps*. All the rhythmic lizards displayed relatively well-synchronised sinusoidal rhythms of ∼10% reflectance amplitude, with a maximum reflectance (i.e. maximum skin lightening) reached in late evening and a minimum reflectance (i.e. maximum skin darkening) reached in late morning. Skin darkening is caused by the dispersion of melanosomes (melanin-bearing organelles) within melanophores, whereas skin lightening results from their aggregation around the perinuclear region [1], [2]. In addition, iridosomes (also called reflecting platelets) contained in iridophores maximise the skin darkening when aggregated and the skin lightening when expanded [30]. Dispersion of dermal and epidermal melanosomes and aggregation of reflecting platelets are effected by the melanocyte-stimulating hormone (MSH) released from the pituitary. On the other hand, aggregation of only dermal melanosomes is stimulated by the release of melatonin from the pineal gland during darkness [30]. The observed rhythmicity in skin reflectance could thus be due to a rhythmic coordination of the release of MSH and melatonin by these endocrine glands both located in the brain. However, it is not clear whether MSH is released during darkness; therefore this rhythmicity could be mainly due to the rhythmic release of melatonin. Moreover, other hormones such as catecholamines are known to affect chromatophores [30]; the mechanisms underlying the circadian colour change under constant darkness may thus involve other effectors and further research would be needed to clarify these hormonal processes.

The endogenous rhythm observed in the lizards’ dorsal skin reflectance was affected by the light/dark cycles. First, the rhythm shifted in time in response to photoperiod, reaching a maximum during the dark phase (approximately 5 hours after the lights went out) and a minimum during the light phase irrespective of the length of the light or dark phase. The shape of individual rhythms varied, but only in the transition timing between extreme values and not in amplitude, which could suggest a similar mechanism of pigment migration occurring within chromatophores in the presence or absence of light. Under a 12∶12 photoperiod, in addition to the shift in time, we observed that the reflectance oscillations from one extreme to the other were not gradual but abrupt, indicating that the light cue triggered the colour change instead of being anticipated by the lizards. On the other hand, under both 6∶18 and 18∶6 photoperiods, the reflectance seemed to increase slightly before the beginning of the dark phases, making it less clear whether the lizards anticipated the beginning of the dark phase due to preliminary acclimation.

Under the 6∶18 photoperiod, the shape of the rhythms seems to indicate that after reaching the maximum, the lizards’ dorsal skin reflectance gradually decreased towards its minimum value because of the increased length of the dark phase. Interestingly, under the 18∶6 photoperiod, the reflectance seemed to remain relatively stable after reaching its minimum value and did not gradually increase. Therefore, the reflectance curves obtained under the 6∶18 and 18∶6 photoperiods do not seem symmetrical, which could suggest that the aggregated state of melanosomes is either more energy-demanding than the dispersed state or more difficult because of physiological constraints. A more costly aggregation state would seem relatively surprising since the lizards are in a rest state during dark phases. Previous investigations on the costs of these two processes provided different conclusions, some considering that pigment aggregation is indeed more costly [31] whereas others found that the dispersion process was more energy-demanding [32]. In any case, the fact that the lizards’ circadian pigmentation rhythm could adjust to the different photoperiods, even more extreme than in the wild (the light phase ranging from 10.5 to 13.5 h in the region of Alice Springs), shows the flexibility of their endogenous pigmentation rhythm, allowing them to interact adaptively with environmental variation.

Light is the most conspicuous entrainment cue and its role in stimulating physiological colour change has been extensively studied [14], [17], [33], [34]. Light transduction is mediated by photoreceptors that can be extraocular [35], [36]. In particular, the pineal gland has been reported to be involved in the circadian colour change of the lizard *A. carolinensis*, through the rhythmic release of melatonin synchronised with light/dark cycles [22], [23], [29], [37]. However, again, it is likely that the circadian pigmentation rhythm involves the action of other effectors, as demonstrated in the ruin lizard *Podarcis sicula* [38], [39].

Reflectance variations were not equal across the whole spectrum. They primarily occurred between 400 and 1200 nm, with maximum change around 700 to 900 nm and relatively little change in the UV and NIR beyond 1400 nm. The total reflectance in UV-Vis in relation to NIR was higher during dark phases compared to light phases. While *P. vitticeps* may modify both visible (UV-Vis range) and NIR reflectance to accommodate requirements of signalling or camouflage and thermoregulation during light phases, they are in a rest state during dark phases and have little need to display colour change for other purposes, which may explain their higher proportion of UV-Vis reflectance. This variation in the proportion of UV-Vis reflectance may be a function of a change in the spacing of reflecting platelets in iridophores, in combination with melanosome dispersion. Light could act as a stimulus for the pituitary to release MSH, resulting in the dispersion of melanosomes and the aggregation of reflecting platelets. The aggregated reflecting platelets could thus be associated with a decrease in the proportion of UV-Vis reflectance in relation to NIR reflectance, although the mechanisms moderating NIR reflectance are currently unknown.

The functional significance of circadian pigmentation rhythms has not been extensively studied. In particular, due to the lack of information on the environmental entrainment cues, the expression of these rhythms under various environmental conditions and the relationships between intracellular mechanisms and colouration at the organismal level remain unclear [14]. A few studies have hypothesised that circadian colour change is displayed for protection from the damaging effects of UV radiation, suggesting that during the day, the skin darkening results in an increased absorption of UV radiation by the superficial tissues of the skin and a decreased transmission of UV radiation to deeper tissues [14], [40]. This hypothesis is supported by numerous observations in crabs and amphibians of chromatophores expanding in response to UV radiation [12], [40], [41]. However, the bearded dragon *P. vitticeps*, like many other species of lizard, possesses a black peritoneum which ensures protection of deeper tissues against damage from near-UV light [42]. Therefore this hypothesis seems less likely to explain the decrease in skin reflectance observed in this study. Since the experimental room was maintained at a constant temperature of ∼30°C during experiments, the displayed rhythms are unlikely to be associated with temperature variation. However, *P. vitticeps* has a preferred body temperature of ∼35°C [43] and uses colour change for thermoregulation in the wild where the temperature varies [44]. As discussed by Coohill *et al.* (1970), these circadian reflectance changes could aid in regulating body temperature, the reflectance decreasing during the day to increase the amount of heat absorbed and help the body warm up [40]. Such a function could be tested by carrying out similar experiments at different temperatures. Further studies are thus needed to clarify the function of circadian colour change and determine whether it is more than just a physiological consequence of having the ability to change colour.

Colour change may be induced by aperiodic environmental events and serve other purposes such as signalling and background matching [9]. Therefore, animals may experience multiple requirements of colour change that may conflict with each other. As a consequence, endogenous time-keeping systems need to possess both stability to adjust to rhythmic environmental events and flexibility to allow the animal to interact adaptively with aperiodic changes [45]. Combining these requirements seems possible thanks to the multioscillatory nature of endogenous rhythms [19], [45]. In particular, this study demonstrates that the expression of the endogenous rhythm is labile and can be modified quickly by exogenous cues. The reversibility of these modifications indicates that short-term environmental events do not substantially modify the rhythm and that notable and prolonged modifications may require a longer entrainment process. However, to confirm the entrainment of the rhythm of colour change by the light/dark cycles, it would be useful to conduct an experiment using the LD 12∶12 regimen with the light phase starting earlier or later to study only the shift of the pigmentation rhythm in time and not its adaptability to shorter or longer light and dark phases.

Three days of acclimation were used in this study and seemed sufficient for a proportion of lizards to synchronise their rhythm; however, it is possible that the other tested lizards would have displayed significant rhythmicity after a longer preliminary period of habituation to the experimental photoperiod. Indeed, these lizards have been captive for a year and a half and constantly exposed to a 12∶12 photoperiodic regimen (except during hibernation); therefore, some of them may have become less responsive to light cues. It should also be noted that in the wild, the transitions between day and night are gradual; therefore switching abruptly from one phase to another is unnatural for the lizards and some of them may need a longer time interval to react to these sudden light changes. It would be informative to use a longer period of acclimation and measure the reflectance from the beginning of the acclimation to monitor the entrainment by light/dark cycles. Additionally, although we tried to avoid disturbing the lizards as much as possible during reflectance measurements, some of them may have reacted more to the presence of the experimenter and to the disturbance caused by the probe placed against their skin for the reflectance readings. They may have thus exhibited punctuated colour changes, generating some artefacts. Therefore, repeating these experiments on a larger group of lizards recently taken from the wild would help to confirm the present results. Nevertheless, this study clearly demonstrates the occurrence of a circadian colour change in the bearded dragon *P. vitticeps* that can be entrained by cyclic environmental factors, such as light/dark cycles, and interact with punctuated colour changes induced by short, aperiodic events, such as inter- or intra-specific signalling.

## Supporting Information

Figure S1 **Dorsal skin reflectance of a lizard placed under LD 12∶12, measured for 24 hours.** Reflectance (expressed in %) was measured from 300 to 2150 nm. Curves in orange shades were obtained at 3-hour intervals during the light phase (from 07∶00 to 19∶00) and curves in blue shades were obtained at 3-hour intervals during the dark phase (from 19∶00 to 07∶00).
(TIF)

Figure S2 **Time series of average dorsal skin reflectance for a rhythmic lizard.** Reflectance (expressed in % and averaged for 300–2150 nm) was calculated for a lizard displaying significant rhythmicity under all four photoperiodic regimens: DD, LD 12∶12, LD 6∶18 and LD 18∶6. The reflectance curves show shifts in time and changes in the shape due to the photoperiod, but no significant variation in average reflectance and amplitude of reflectance.
(TIF)

Figure S3 **Time series of** ***δ*** **for rhythmic lizards under the four photoperiodic regimens.** *δ* represents the standardised difference between the total reflectance in near-infrared (700–2150 nm) and the total reflectance in ultraviolet-visible (300–700 nm). *δ* was calculated for lizards displaying significant rhythmicity under DD, LD 12∶12, LD 6∶18 and LD 18∶6. The curves show no significant variation in average value and amplitude.
(TIF)

Table S1 ***p*** **values provided by the Tukey’s HSD test for post hoc comparisons.** Post hoc comparisons were performed between photoperiodic regimens for parameters showing significant variation due to the photoperiod (i.e. parameters for which the one-way ANOVA provided a *p* value smaller than 0.05). *: *p*<0.05; **: *p*<0.01 and ***: *p*<0.001.
(DOC)
