# Supplementary figures and images for: Cyclic Colour Change in the Bearded Dragon Pogona vitticeps under Different Photoperiods
Source: PLoS One. 2014 Oct 29;9(10):e111504. doi: 10.1371/journal.pone.0111504 (PMC4213017; doi:10.1371/journal.pone.0111504)

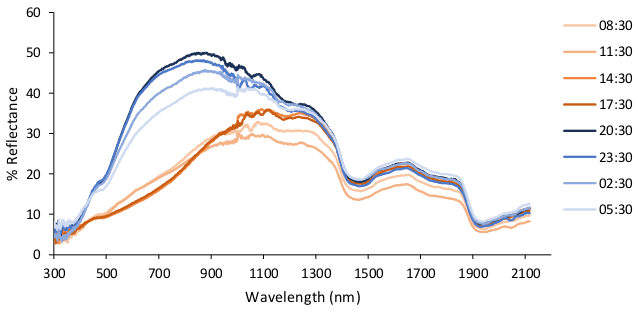

Supplement: Figure S1 — Dorsal skin reflectance of a lizard placed under LD 12∶12, measured for 24 hours. Reflectance (expressed in %) was measured from 300 to 2150 nm. Curves in orange shades were obtained at 3-hour intervals during the light phase (from 07∶00 to 19∶00) and curves in blue shades were obtained at 3-hour intervals during the dark phase (from 19∶00 to 07∶00). (TIF) [file pone.0111504.s001.tif]

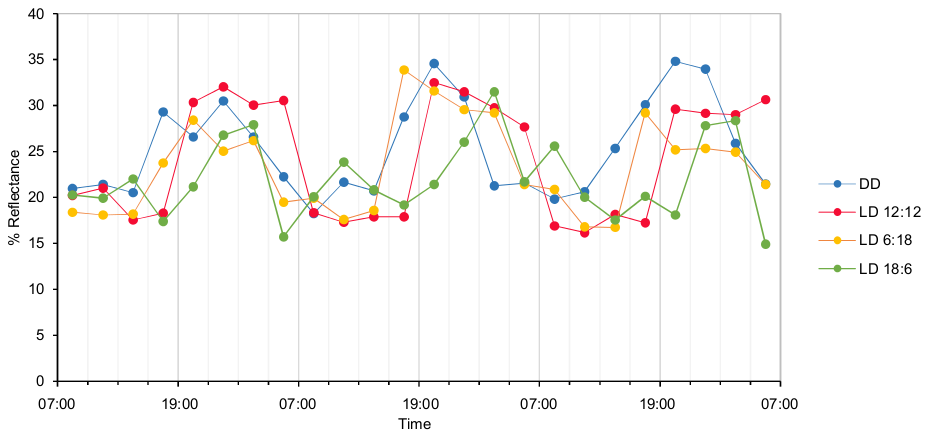

Supplement: Figure S2 — Time series of average dorsal skin reflectance for a rhythmic lizard. Reflectance (expressed in % and averaged for 300–2150 nm) was calculated for a lizard displaying significant rhythmicity under all four photoperiodic regimens: DD, LD 12∶12, LD 6∶18 and LD 18∶6. The reflectance curves show shifts in time and changes in the shape due to the photoperiod, but no significant variation in average reflectance and amplitude of reflectance. (TIF) [file pone.0111504.s002.tif]

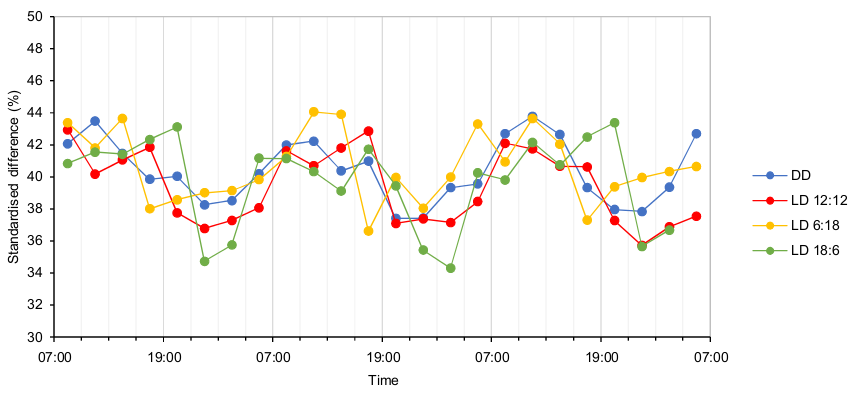

Supplement: Figure S3 — Time series of δ for rhythmic lizards under the four photoperiodic regimens. δ represents the standardised difference between the total reflectance in near-infrared (700–2150 nm) and the total reflectance in ultraviolet-visible (300–700 nm). δ was calculated for lizards displaying significant rhythmicity under DD, LD 12∶12, LD 6∶18 and LD 18∶6. The curves show no significant variation in average value and amplitude. (TIF) [file pone.0111504.s003.tif]
