# Supplementary material for: Cyclic Colour Change in the Bearded Dragon Pogona vitticeps under Different Photoperiods
Source: PLoS One. 2014 Oct 29;9(10):e111504. doi: 10.1371/journal.pone.0111504 (PMC4213017; doi:10.1371/journal.pone.0111504)
Supplement: Table S1 — p values provided by the Tukey’s HSD test for post hoc comparisons. Post hoc comparisons were performed between photoperiodic regimens for parameters showing significant variation due to the photoperiod (i.e. parameters for which the one-way ANOVA provided a p value smaller than 0.05). *: p<0.05; **: p<0.01 and ***: p<0.001. (DOC) [file pone.0111504.s004.doc]

| Tukey's HSD test | | | | | | |
| --- | --- | --- | --- | --- | --- | --- |
| Parameter | 12:12 vs DD | 18:6 vs DD | 6:18 vs DD | 18:6 vs 12:12 | 6:18 vs 12:12 | 6:18 vs 18:6 |
| *τ*(*φ*) | * | ** | 1.00 | 0.35 | * | ** |
| *τ_1_* | 0.95 | 0.19 | * | * | 0.08 | *** |
| *τ_2_* | 0.92 | 0.23 | ** | * | ** | *** |
| *τ_+_* | 0.98 | 0.14 | *** | *** | 0.06 | *** |
| *τ-* | 0.85 | 0.58 | 0.08 | 0.13 | 0.23 | ** |
